# Dynamic expression of *Lgr6* in the developing and mature mouse cochlea

**DOI:** 10.3389/fncel.2015.00165

**Published:** 2015-05-12

**Authors:** Yanping Zhang, Yan Chen, Wenli Ni, Luo Guo, Xiaoling Lu, Liman Liu, Wen Li, Shan Sun, Lei Wang, Huawei Li

**Affiliations:** ^1^Research Center, Affiliated Eye and ENT Hospital of Fudan UniversityShanghai, China; ^2^Key Laboratory of Hearing Medicine, Ministry of Health, Affiliated Eye and ENT Hospital of Fudan UniversityShanghai, China; ^3^Institutes of Biomedical Sciences, Fudan UniversityShanghai, China; ^4^Department of Otorhinolaryngology, Affiliated Eye and ENT Hospital of Fudan UniversityShanghai, China; ^5^The State Key Laboratory of Medical Neurobiology, The Institutes of Brain Science and the Collaborative Innovation Center for Brain Science, Fudan UniversityShanghai, China

**Keywords:** Wnt, regeneration, hair cells, Lgr6, inner pillar cell, inner border cell, hair cell progenitor

## Abstract

The Wnt/β-catenin signaling pathway plays important roles in mammalian inner ear development. *Lgr5*, one of the downstream target genes of the Wnt/β-catenin signaling pathway, has been reported to be a marker for inner ear hair cell progenitors. *Lgr6* shares approximately 50% sequence homology with *Lgr5* and has been identified as a stem cell marker in several organs. However, the detailed expression profiles of *Lgr6* have not yet been investigated in the mouse inner ear. Here, we first used Lgr6-EGFP-Ires-CreERT2 mice to examine the spatiotemporal expression of Lgr6 protein in the cochlear duct during embryonic and postnatal development. Lgr6-EGFP was first observed in one row of prosensory cells in the middle and basal turn at embryonic day 15.5 (E15.5). From E18.5 to postnatal day 3 (P3), the expression of Lgr6-EGFP was restricted to the inner pillar cells (IPCs). From P7 to P15, the Lgr6-EGFP expression level gradually decreased in the IPCs and gradually increased in the inner border cells (IBCs). At P20, Lgr6-EGFP was only expressed in the IBCs, and by P30 Lgr6-EGFP expression had completely disappeared. Next, we demonstrated that Wnt/β-catenin signaling is required to maintain the Lgr6-EGFP expression *in vitro*. Finally, we demonstrated that the Lgr6-EGFP-positive cells isolated by flow cytometry could differentiate into myosin 7a-positive hair cells after 10 days in-culture, and this suggests that the Lgr6-positive cells might serve as the hair cell progenitor cells in the cochlea.

## Introduction

The mammalian inner ear cochlea is a very sophisticated organ. The organ of Corti in the inner ear is responsible for hearing in mammals and contains two types of mechanosensory hair cells (inner hair cells and outer hair cells) and six types of supporting cells [Deiters' cells, Hensen's cells, Claudius' cells, pillar cells, inner phalangeal cells, and inner border cells (IBCs)] (Chai et al., 2012). Cell proliferation and differentiation are coordinated in the development of the organ of Corti, but many molecular mechanisms involved in regulating this proliferation and differentiation remain undiscovered (Cremisi et al., 2003; Pagano and Jackson, 2004). During the development of the mouse cochlea, the otic placode—which will give rise to the auditory and vestibular sensory organs of the inner ear (Fritzsch et al., 1998)—forms on embryonic day 8.5 (E8.5). The placode invaginates to form the otocyst on E11. By this time, the regions that will give rise to sensory neuroepithelia have already been specified (Morsli et al., 1998). At E12, the progenitors of hair cells and supporting cells are still dividing (Ruben, 1967). At E12.5, apical progenitors begin to exit the cell cycle, and this is followed by the progenitor cells in the base of the cochlea. By E14, all progenitor cells of the inner ear have exited the cell cycle (Ruben, 1967; Lee et al., 2006). Hair cell differentiation first appears in the mid-basal region of the cochlea at E13.5 and spreads bidirectionally during the development of the organ of Corti (Chen et al., 2002). Hair cell differentiation finishes between E17 and E18 (Sher, 1971; Li and Ruben, 1979; Lim and Anniko, 1985; Chen et al., 2002).

The Wnt/β-catenin signaling pathway is reported to play important roles in mammalian cochlear development and hair cell regeneration by regulating both proliferation and differentiation of prosensory cells. The Wnt/β-catenin signaling pathway is also critical for otocyst induction, dorsal patterning of the otocyst, and the eventual formation of the vestibular organs (Hollyday et al., 1995; Jasoni et al., 1999; Riccomagno et al., 2005; Lillevali et al., 2006; Ohyama et al., 2006; Rakowiecki and Epstein, 2013). Wnt/β-catenin signaling also plays a key role in hair cell formation in the cochlea, and this is clearly demonstrated by the inability of hair cells to differentiate from sensory progenitors when Wnt/β-catenin is knocked out (Jacques et al., 2012; Shi et al., 2014).

Recently, the Wnt/β-catenin downstream target genes *Lgr5* and *Axin2* have been reported to be markers for inner ear hair cell progenitors. The Lgr5-positive hair cell progenitors can self-renew to regenerate hair cells after isolation by flow cytometry *in vitro*, and they can also spontaneously regenerate hair cells after hair cell loss in neonatal mouse cochleae *in vivo* (Chai et al., 2011, 2012; Shi et al., 2012, 2013; Jan et al., 2013; Cox et al., 2014). Recent studies also showed that Wnt/β-catenin signaling plays dual roles in controlling the proliferation and differentiation of hair cell progenitor cells (Jacques et al., 2012; Shi et al., 2014). Lgr6 is a member of the leucine-rich repeat-containing G-protein-coupled receptors (LGRs) (Barker and Clevers, 2010). In other organs, including the skin, taste buds, and lungs, Lgr6 has been identified as a stem cell marker (Snippert et al., 2010; Oeztuerk-Winder et al., 2012; Ren et al., 2014), and these Lgr6-positive stem cells have been reported to be involved in wound repair and hair follicle development (Barker and Clevers, 2010; Snippert et al., 2010; Leushacke and Barker, 2012). Lgr6 expression is sometimes up-regulated in gastric cancer, and Lgr6 expression is significantly correlated with patient survival; patients with Lgr6-positive tumors tend to live longer than patients with Lgr6-negative tumors (Krejs, 2010; Garlipp et al., 2011).

Lgr6 shares approximately 50% sequence homology with Lgr5, which is a marker of Wnt-regulated hair cell progenitor cells in the postnatal mouse cochleae. The expression pattern and characterization of Lgr5 have been well studied in the mouse inner ear (Chai et al., 2011, 2012; Shi et al., 2012, 2013; Jan et al., 2013; Cox et al., 2014). At E15.5, Lgr5 is expressed in the whole prosensory region of the cochlear duct, and the expression continues to decrease during development. From E18.5 to neonatal ages, Lgr5 expression is restricted to the third row of Deiters' cells, inner pillar cells (IPCs), medial inner phalangeal cells, and the lateral GER (greater epithelium region). By P30, Lgr5 expression is only detectable in the third row of Deiters' cells (Chai et al., 2011). However, the detailed expression profiles of the homologous Lgr6 protein during development have not yet been investigated in the mouse inner ear. In the gastrointestinal and integumentary systems, Lgr5 expression is regulated by Wnt signaling (Jaks et al., 2008; Ootani et al., 2009). In the inner ear, Wnt activation increases Lgr5 expression, and Wnt inhibition decreases Lgr5 expression (Chai et al., 2011, 2012; Shi et al., 2012, 2013). However, whether Wnt signaling can regulate the expression of Lgr6 has remained unknown. In mouse cochleae, the Lgr5-positive cells can regenerate hair cells both *in vivo* and *in vitro* and thus, serve as the inner ear hair cell progenitor cells (Chai et al., 2012; Shi et al., 2012, 2013; Cox et al., 2014; Li et al., 2015). However, the characterization of the Lgr6-positive cells has not been investigated in the mouse inner ear.

In this study, we first utilized transgenic Lgr6-EGFP-Ires-CreERT2 reporter mice to characterize the spatiotemporal expression of Lgr6 in the embryonic and postnatal mammalian cochlear duct. Next, we showed that Wnt signaling is required to maintain the expression of Lgr6. Last, we isolated the Lgr6-positive cells by flow cytometry and demonstrated that Lgr6-positive cells could differentiate into hair cells *in vitro*.

## Methods

### Animals

Lgr6-EGFP-Ires-CreERT2 mice in a C57BL/6J background were purchased from the Jackson Laboratory (stock no. 016934). These mice were generated by homologous recombination in embryonic supporting cells that placed an EGFP-Ires-CreERT2 cassette at the transcriptional start site of the *Lgr6* gene. Insertion of the EGFP-Ires-CreERT2 into the transcriptional start site of the gene enabled green fluorescent labeling of cells that normally express Lgr6. All of the transgenic mice used for our research were heterozygotes. All animal procedures were performed according to protocols approved by the Animal Care and Use Committee of Fudan University and were consistent with the National Institutes of Health Guide for the Care and Use of Laboratory Animals. All efforts were made to minimize the number of animals used and to prevent their suffering.

### Genotyping, RT-PCR, and qPCR

Genomic DNA was isolated from transgenic mouse tails using a DNA extraction kit (pc 3202, Biomed) according to the manufacturer's instructions. We used the following genotyping primers: wild-type Lgr6 (forward) CTG TGG CTT TGC GCT GTG; (reverse) AAG GGC ACC AAA CGA GTG T; mutant Lgr6 (forward) GCC CAC CGA CGG CGC AGC CC; (reverse) GCT GAA CTT GTG CCG TTT A.

The total RNA from the cochlear sensory epithelium was processed with an RNeasy micro kit (Cat. #74004, Qiagen). cDNA was synthesized from 1 μg total RNA by reverse transcription using random primers (Promega) and Superscript III reverse transcriptase (Life Technologies). Quantitative real-time PCR (qPCR) was performed using SYBR Green PCR Master Mix (Life Technologies) on an AB 7500 Real-Time PCR System (Life Technologies). Each PCR reaction was carried out in triplicate, and the relative quantification of gene expression was analyzed using the ΔΔCT method with GAPDH as the endogenous reference. Primer pairs for the qPCR were designed using the online Primer3 software. *Lgr6*: (forward) GTA TGA ACA ACC TCA CGG AGC; (reverse) TTG GAG GCC AGA GAA TGC C; *Sox2*: (forward) ATG AAC GGC TGG AGC AAC GGC A; (reverse) TCA CAT GTG CGA CAG GGG CAG T; *GAPDH*: (forward) AAC GGG AAG CCC ATC ACC ATC TT; (reverse) CAG CCT TGG CAG CAC CAG TGG; *Lgr5*: (forward) TCT TCA CCT CCT ACC TGG ACC T; (reverse) GGC GTA GTC TGC TAT GTG GTG T; *Brn3.1*: (forward) CCC AAA TTC TCC AGC CTA CAC; (reverse) GGC GAG ATG TGC TCA AGT AAG T.

### Cryosectioning

Heads from E15.5 mice and the otic bullae from E18.5–postnatal day 3 (P3) mice were isolated and fixed with 4% paraformaldehyde (Sigma-Aldrich) in 0.01 M phosphate-buffered saline (PBS, pH 7.4) at 4°C overnight. For P7 and older animals, decalcification was performed with 10% EDTA in PBS for 1–3 days at 4°C. Samples were immersed in 15% and then 30% sucrose in PBS at 4°C overnight, and then embedded in OCT compound (Sakura Finetek) at 4°C overnight. Serial frozen sections of 10–12 μm thickness were made with a Leica CM3050 cryostat (Leica).

### Cell sorting by flow cytometry

Cochleae from P3 Lgr6-EGFP-Ires-CreERT2 mice were dissected out and the stria vascularis and spiral ganglia were removed. The cochleae were incubated in 0.125% trypsin (Invitrogen) at 37°C for 8 min, and the same volume of 10 mg/mL trypsin inhibitor (Worthington Biochem) was added. Following trituration, cells were passed through a 40 μm filter (BD Biosciences) and labeled with 1 μg/mL propidium iodide (Sigma) to remove the dead cells. Wild-type cochleae were used to determine the background labeling levels in each sorting. The dissociated cells were sorted on a MoFlo® SX FACS cytometer (Beckman Coulter) using the channels for GFP, and the positive fractions were collected. We consistently achieved over 90% cell viability and over 95% purity for sorted cells. The purity of the sorted cells was assessed by re-sort analysis, immunohistochemistry, and quantitative RT-PCR. For staining, sorted cells were plated on fibronectin-coated slides (Sigma) and incubated for 1 h at 37°C before fixation and immunohistochemistry.

### Culture of sorted cells

Flow cytometry-isolated Lgr6-positive cells (2000 cells with a density of 20 cells/μL) were plated on a laminin-coated 4-well dish and cultured for 10 days in DMEM/F12 media with 2% B27, 1% N2, EGF (20 ng/mL, Chemicon), bFGF (10 ng/mL, NIH), IGF-1 (50 ng/mL, Chemicon), and heparin sulfate (50 ng/mL, Sigma). The cells were fixed and immunostained for hair cell markers after 10 days of culture.

### Tissue cultures

P1 mice were sacrificed and their cochleae were carefully dissected to separate the organs of Corti from the spiral ganglia, the stria vascularis, and Reissner membranes under sterile conditions with fine forceps. Whole-mount cochleae were then transferred onto 10-mm coverslips (Fisher Scientific) pre-coated with poly-L-lysine (Sigma-Aldrich). Whole organs were cultured in DMEM/F12 (Invitrogen) supplemented with N2 (Invitrogen), B27 (Invitrogen), and ampicillin (50 μg/mL; Sigma-Aldrich) in four-well Petri dishes (Greiner Bio-one). After 12 h, Bio (5 μM, Sigma) or IWP-2 (25 mM, Sigma) was added to the culture medium for 3 days. Culture media were replenished every day. After this final culture, the basal membranes were fixed with 4% paraformaldehyde for 30 min at room temperature.

### Immunofluorescence, image acquisition, and image analysis

Immunofluorescence was performed as reported previously (Jiang et al., 2014). Fixed tissues were blocked with 10% donkey serum in 10 mM PBS (pH 7.4) with 1% Triton X-100 (Sangon Biotech) for 1 h at room temperature and then incubated with primary antibodies overnight at 4°C in a humidified chamber. The following day, tissues were rinsed with PBS and then incubated with secondary antibodies for 1 h at room temperature. After washing with PBS, tissues were mounted in antifade fluorescence mounting medium (DAKO) and coverslipped. The primary antibodies included anti-Lgr6 (1:500 dilution, Santa Cruz Biotechnology), anti-myosin 7a (1:1000 dilution, Proteus Bioscience), anti-GFP (1:1000 dilution, Abcam), and anti-Sox2 (1:500 dilution, Santa Cruz Biotechnology). The secondary antibodies were conjugated with FITC, Cy3, or Cy5 (1:500 dilution, Jackson ImmunoResearch). The nuclei were stained with 4,6-diamidino-2-phenylindole (DAPI, 1:800 dilution, Sigma-Aldrich) for 15 min at room temperature. Negative control experiments were performed as above by omitting the primary antibodies. Cochleae were dissected into the apical, middle, and basal turns, and photographs were taken using a Leica SP5 confocal fluorescence microscope (Leica) and analyzed with Photoshop CS4 (Adobe Systems).

### Auditory brainstem responses

The auditory brainstem response (ABR) was measured to determine the hearing threshold of the mice. P30 mice were anaesthetized with ketamine (100 mg/kg) and xylazine (25 mg/kg; i.p.) and then placed on a thermostatic heating pad in a sound-attenuating chamber to maintain their body temperatures at 38°C. Electrodes were placed on the scalp of the mouse to record the electrical activity of the brain in response to sound. Four frequencies (8, 16, 24, and 32 kHz) were assessed with a TDT system 3 device (Tucker Davies Technologies).

### Cell counts

To quantify the Lgr6-positive cells in the three turns of the cochlea, we imaged the entire cochlea using a 40× objective and counted the EGFP-positive cells. For all experiments, only one cochlea from each mouse was used for immunofluorescence and quantification. Thus, *n* represents the number of mice examined.

### Statistical analysis

Statistical analyses were conducted using Microsoft Excel (Microsoft) and GraphPad Prism v5.0.3. The data were expressed as the mean ± SD. Immunofluorescence analysis was performed with a two-tailed, unpaired Student's *t*-test when comparing two groups or with a one-way ANOVA followed by a Dunnett's multiple comparisons test when comparing more than two groups. *P* < 0.05 was considered statistically significant.

## Results

### Lgr6 expression in the embryonic cochlear duct

**Figure 1:**
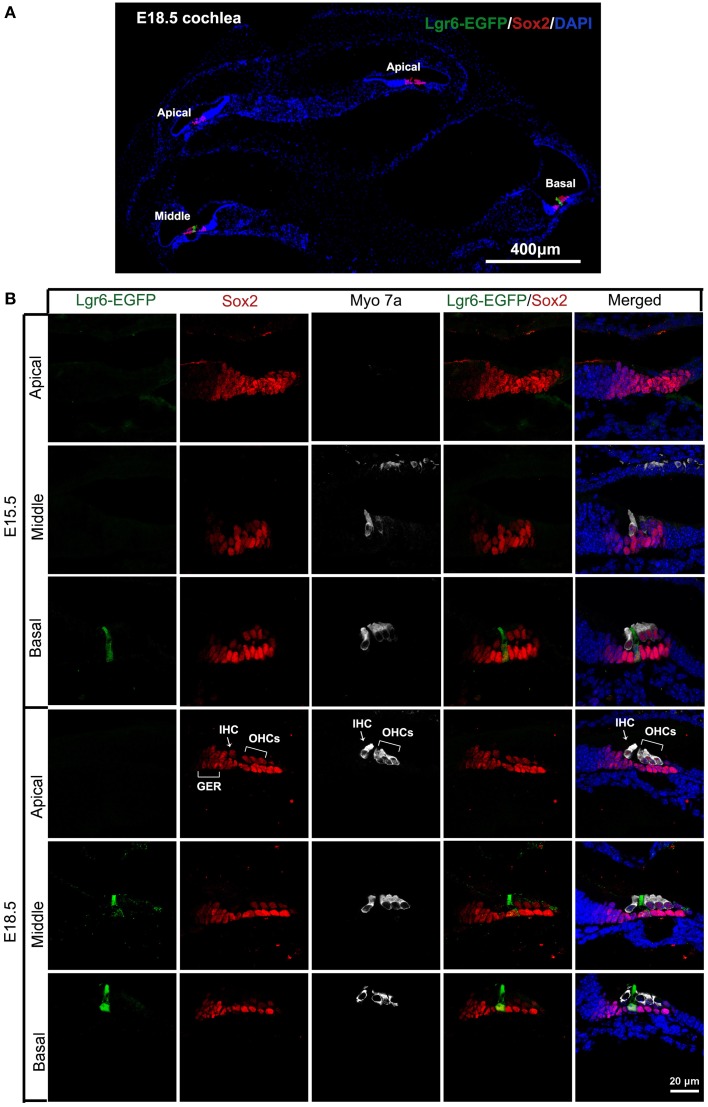
**Expression of Lgr6-EGFP in cochlear cryosections at E15.5 and E18.5. (A)** At low magnification, expression of Lgr6-EGFP at E18.5 was restricted to one supporting cell in the middle and basal turns, and no expression was seen in the apical turn. **(B)** Lgr6-EGFP was expressed in one supporting cell in the middle and basal turns of the organ of Corti at E15.5. At E18.5, Lgr6-EGFP was expressed in the inner pillar cells in the middle and basal turns. IHC, inner hair cell; OHCs, outer hair cells; GER, greater epithelium region.

**Figure 2:**
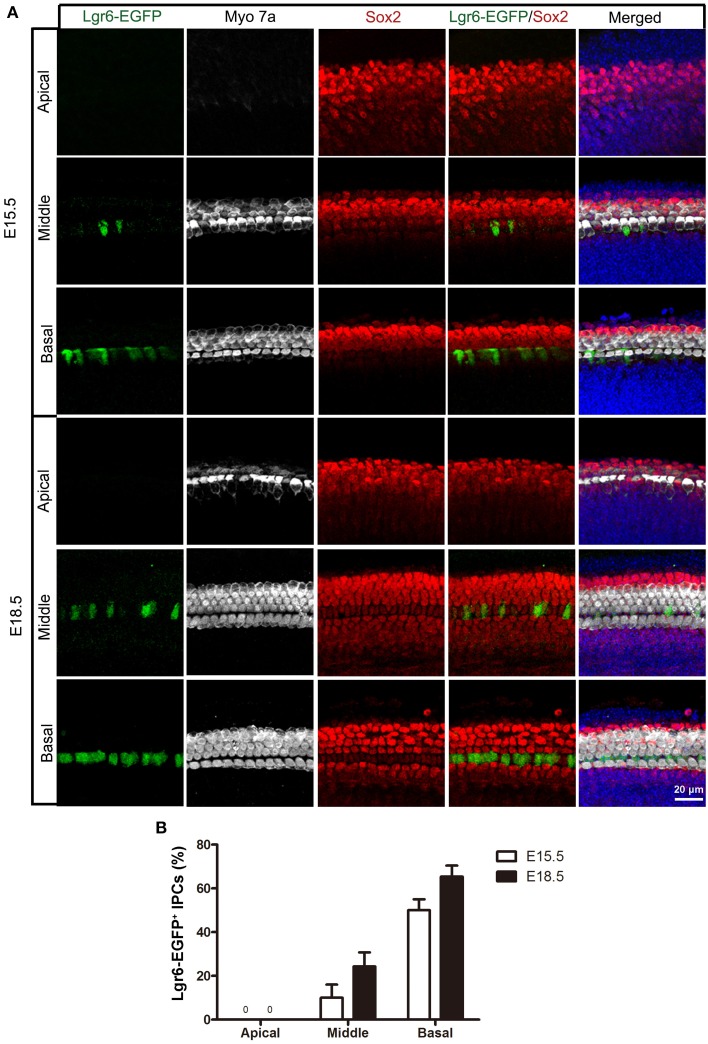
**Lgr6-EGFP expression in the whole mount cochleae of mouse embryos at E15.5 and E18.5. (A)** Lgr6-EGFP was expressed in one row of supporting cells in the middle and basal turns of the organ of Corti at E15.5 and E18.5. There was no Lgr6-EGFP expression in the apical turn. **(B)** The percentage of Lgr6-EGFP-positive IPCs at E15.5 and E18.5. The Lgr6-EGFP expression increased in both the middle and basal turns of the organ of Corti from E15.5 to E18.5 (*n* = 4). IPCs, inner pillar cells.

We first investigated the expression pattern of the Wnt downstream target gene *Lgr6*. Here we took advantage of the Lgr6-EGFP-Ires-CreERT2 transgenic mice in which EGFP-Ires-CreERT2 is inserted into the transcriptional start site of the *Lgr6* gene. The cells in these mice that normally express Lgr6 are labeled with green fluorescent protein. At E13.5, we did not observe any Lgr6-EGFP-positive cells in the organ of Corti (data not shown). Two days later, at E15.5, we detected Lgr6-EGFP expression in one row of Sox2-positive progenitor cells, and the Lgr6 expression appeared as a gradient from the base to the apex in the cochlear duct floor epithelium (Figures 1B, 2A). Lgr6-EGFP was mainly expressed in the middle and basal turns of the organ of Corti, and no Lgr6-EGFP was observed in the apical turn (*n* = 4) (Table 1). Starting at E15.5, myosin 7a, one of the hair cell-specific markers, was expressed in the base of the organ of Corti, and Sox2, the prosensory cell marker, was expressed in the prosensory region that gives rise to the organ of Corti (Kiernan et al., 2005; Dabdoub et al., 2008). In the basal turn, Lgr6-EGFP expression was only seen in one row of prosensory cells between the inner hair cells and outer hair cells (Figure 1B).

**Table 1:** **Quantification of Lgr6-EFGP/Sox2 double-positive inner pillar cells per 100 μm cochlear length at E15.5 and E18.5**.

**Embryonic**	**Apical**	**Middle**	**Basal**
E15.5	0	2.53 ± 0.50	10.13 ± 0.71
E18.5	0	4.12 ± 0.83	12.08 ± 0.50

*All the data are shown as the average ± S.D. n = 4 for all the samples*.

Hair cells complete their differentiation between E17 and E18. We examined the E18.5 cochlear duct and found a similar Lgr6-EGFP expression pattern with a gradient from base to apex as seen at E15.5. Lgr6-EGFP was expressed in the IPCs in the middle and basal turns (Figure 1A), and the Lgr6-EGFP expression was increased compared to E15.5 (*n* = 4) (Figures 1, 2, and Table 1). The percentage of Lgr6-EGFP-positive cells out of the total IPCs was 24.42 ± 4.43% and 65.33 ± 5.13% in the middle and basal turns, respectively. We did not detect any Lgr6-EGFP-positive cells in the apical turn. At this development stage, we detected four rows of myosin 7a-positive hair cells in the organ of Corti (Figure 2A). In the apical and middle turn, Sox2 was expressed in hair cells, the adjacent supporting cells, and the GER medially (Figure 1B). This hair cell-bearing area at this stage is henceforth referred to as the prosensory region. Sox2 levels were down-regulated in more differentiated hair cells in the basal region (Figure 1B), and this down-regulation of Sox2 in hair cells was consistent with previous reports (Hume et al., 2007).

### Lgr6-EGFP expression is restricted to the IPCs in the cochlea at P1 and P3

**Figure 3:**
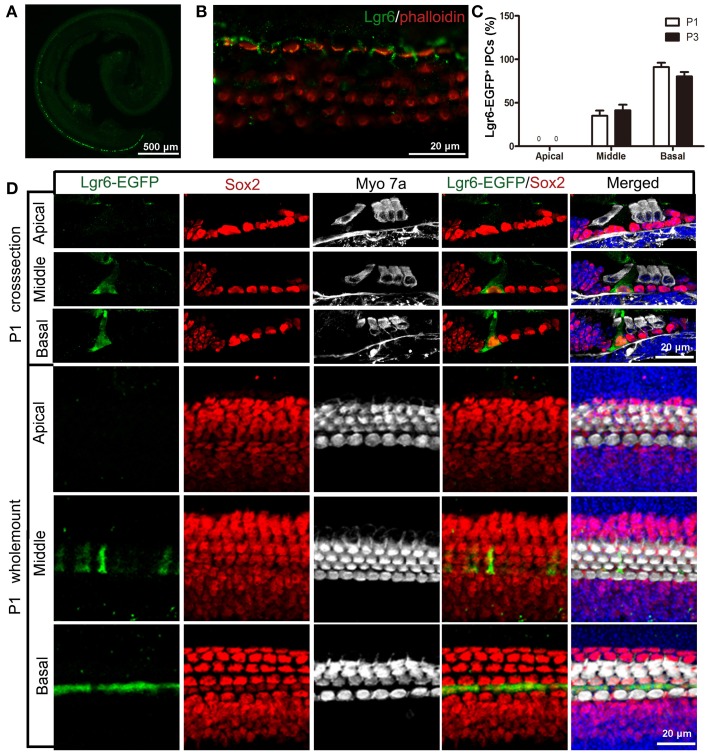
**Lgr6-EGFP expression is restricted to the IPCs in the cochlea at P1. (A)** At low magnification, only one row of Lgr6-EGFP-positive cells was detected, and Lgr6-EGFP was only expressed in the middle and basal turns in the cochlear epithelium at P1. **(B)** Immunostaining with an anti-Lgr6 antibody in wild-type cochlea showed that Lgr6 expression was restricted to the IPCs at P1. **(C)** The percentage of Lgr6-EGFP-positive IPCs (IPCs) at P1 and P3 (*n* = 4). **(D)** At P1, Lgr6-EGFP was expressed only in the IPCs in the middle and basal turns. IPCs, inner pillar cells.

Like the E18.5 cochlear duct, Lgr6-EGFP was also expressed in the IPCs in the middle and basal turns of P1 and P3 cochleae (Figures 3A,C,D and Table 2). There was no Lgr6-EGFP expression in the apical turn. At P1, Lgr6 expression increased in both the middle and basal turns compared to E18.5 (*n* = 4) (Tables 1, 2). To confirm the expression of Lgr6, we stained the P1 cochleae with the Lgr6 antibody and found that the Lgr6 expression detected by immunohistochemical staining was identical to the Lgr6-EGFP reporter expression (Figure 3B). At P3, the Lgr6 expression decreased in both the basal and middle turns of the organ of Corti compared to the expression at P1 (*n* = 4) (Figure 3C, Figure S1, and Table 2).

**Table 2:** **Quantification of Lgr6-EFGP/Sox2 double-positive inner pillar cells per 100 μm cochlear length from P1 to P30**.

**Age**	**Apical**	**Middle**	**Basal**
P1	0	9.67 ± 0.35	15.03 ± 0.25
P3	0	7.03 ± 0.25	12.00 ± 0.30
P7	0	4.57 ± 0.26	10.40 ± 0.31
P10	0	2.20 ± 0.30	8.37 ± 0.25
P15	0	2.11 ± 0.28	7.87 ± 0.18
P20	0	0	0
P30	0	0	0

*All the data are shown as the average ± S.D. n = 4 for all the samples*.

### Lgr6-EGFP expression is restricted to both IPCs and IBCs from P7 to P15

**Figure 4:**
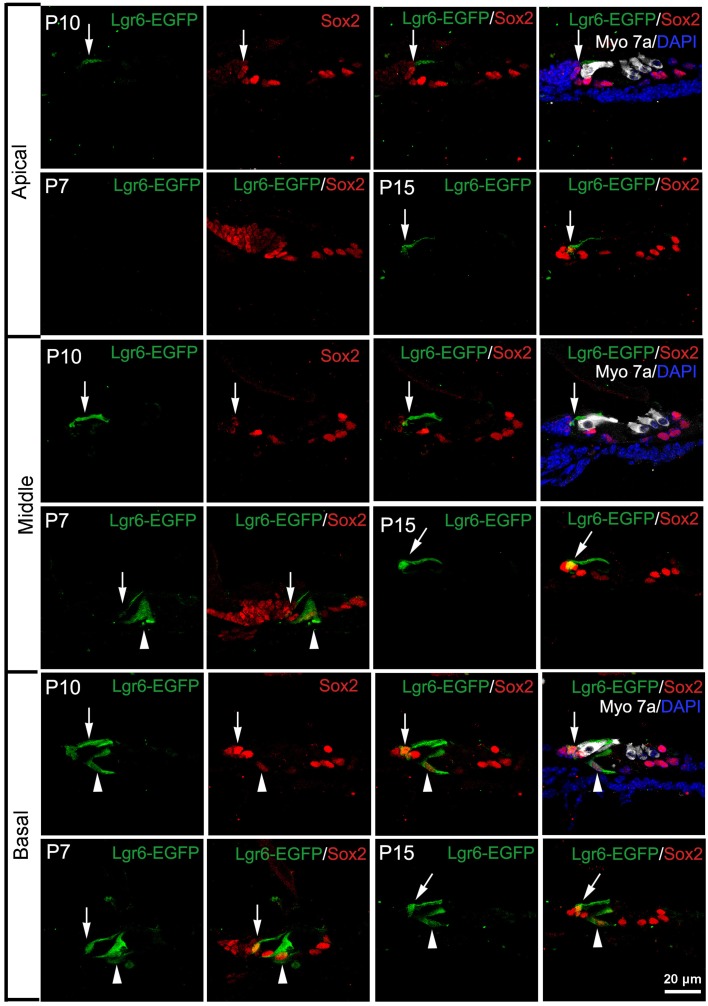
**Lgr6-EGFP expression is restricted to both inner pillar cells (IPCs) and inner border cells (IBCs) from P7 to P15**. At P7, Lgr6-EGFP was expressed both in the IPCs (arrow head) and IBCs (arrow) in the middle and basal turns, and there was no Lgr6-EGFP expression in the apical turn. Between P10 and P15, Lgr6-EGFP was expressed only in the IPCs (arrow head), but in the basal turn Lgr6-EGFP was expressed in both the IPCs (arrow head) and IBCs (arrow).

**Figure 5:**
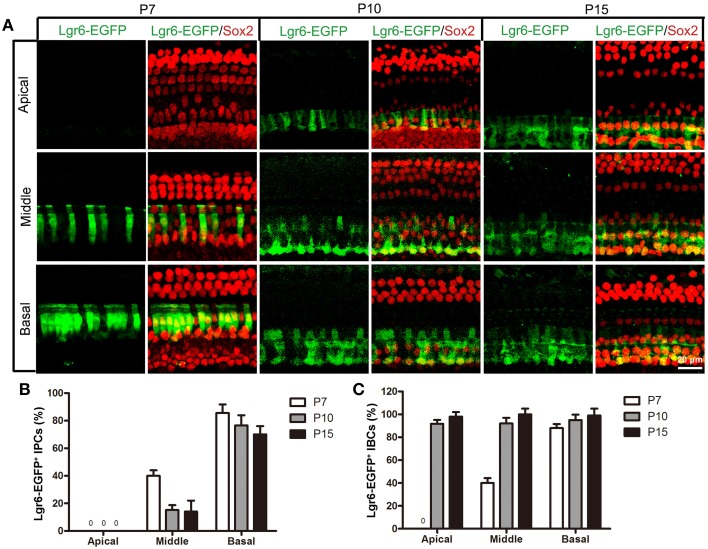
**Lgr6-EGFP expression decreased in the IPCs and increased in the IBCs from P7 to P15. (A)** From P7 to P15, Lgr6-EGFP was expressed in both the IPCs and IBCs in the middle and basal turns. In the apical turn, there was no Lgr6-EGFP expression at P7, and Lgr6-EGFP was only expressed in the IBCs at P10 and P15. **(B)** From P7 to P15, the percentage of Lgr6-EGFP-positive IPCs gradually decreased (*n* = 4), **(C)** From P7 to P15, the percentage of Lgr6-EGFP-positive IBCs gradually increased (*n* = 4). IPCs, inner pillar cells; IBCs, inner border cells.

**Figure 6:**
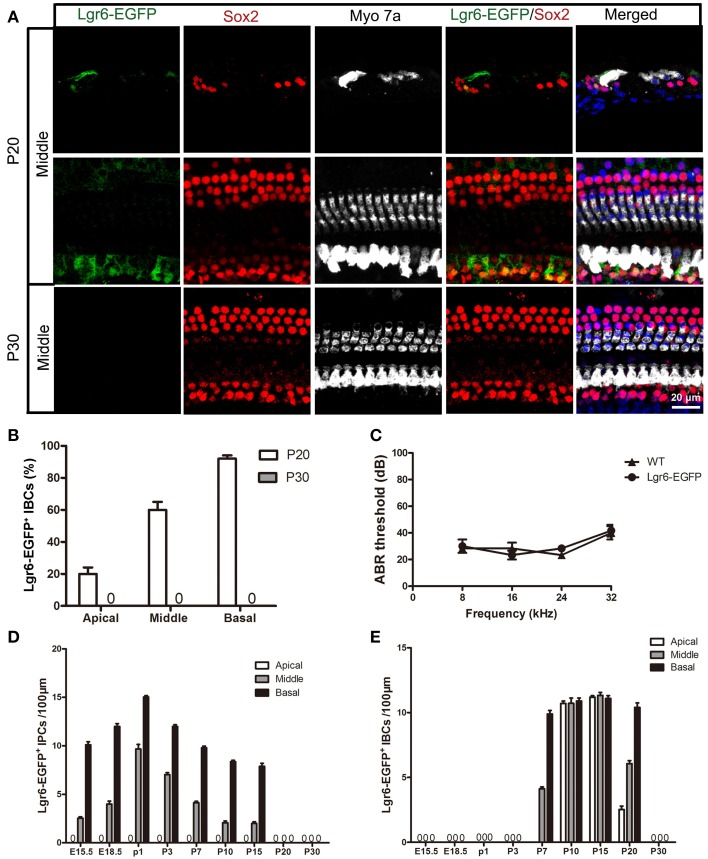
**Lgr6-EGFP expression was restricted to IBCs at P20 and completely disappeared at P30. (A)** Lgr6-EGFP was expressed only in the IBCs at P20, and Lgr6-EGFP expression disappeared at P30. **(B)** Lgr6-EGFP expression decreased gradually from the basal to apical turn at P20 (*n* = 4). **(C)** The P30 heterozygous Lgr6-EGFP-Ires-CreERT2 mice had normal hearing. **(D)** The number of Lgr6-EGFP-positive IPCs per 100 μm cochlear epithelium from E15.5 to P30. The number of Lgr6-EGFP-positive IPCs started to increase at E15.5, peaked at P1, and then gradually decreased and disappeared by P20 (*n* = 4). **(E)** The number of Lgr6-EGFP-positive IBCs per 100 μm cochlear epithelium from E15.5 to P30. The Lgr6-EGFP-positive IBCs first appeared at P7. The number of cells increased and reached a peak at P15 and then decreased and disappeared by P30 (*n* = 4). IPCs, inner pillar cells; IBCs, inner border cells.

Interestingly, at P7 we found that the Lgr6-EGFP expression was not only in the IPCs but also in the IBCs in both the middle and basal turns. There was no EGFP expression in the apical turn. The Lgr6 expression in the IPCs decreased at P7 compared to that of P3 (*n* = 4) (**Figure 6D**, Table 2). The Lgr6 expression in IBCs was similar to that in IPCs (*n* = 4) (Figures 4, 5A,B, 6E and Table 3). The percentage of Lgr6-EGFP-positive IPCs was 40 ± 3.6% and 85 ± 4.7% in the middle and basal turns, respectively. The percentage of Lgr6-EGFP-positive IBCs was 41.5 ± 4.1% and 87.3 ± 3.53% in the middle and basal turns, respectively.

**Table 3:** **Quantification of Lgr6-EFGP/Sox2 double-positive inner border cells per 100 μm cochlear length from P1 to P30**.

**Age**	**Apical**	**Middle**	**Basal**
P1	0	0	0
P3	0	0	0
P7	0	4.30 ± 0.23	9.9 ± 0.31
P10	10.72 ± 0.39	10.78 ± 0.36	10.92 ± 0.19
P15	11.17 ± 0.35	11.25 ± 0.45	11.20 ± 0.15
P20	2.53 ± 0.45	6.07 ± 0.40	10.60 ± 0.25
P30	0	0	0

*All the data are shown as the average ± S.D. n = 4 for all the samples*.

At P10, Lgr6-EGFP expression in the middle and basal turns was similar to P7 (*n* = 4) (Figures 4, 5, 6D,E, Tables 2, 3). The percentage of Lgr6-EGFP-positive IPCs was 15.19 ± 3.56% and 76.53 ± 4.12% in the middle and basal turns, respectively. The percentage of Lgr6-EGFP-positive IBCs was 92.1 ± 4.78% and 95.08 ± 4.61% in the middle and basal turns, respectively. However, Lgr6-EGFP expression also appeared in the IBCs of the apical turn (*n* = 4) (Table 3), and the percentage of Lgr6-EGFP-positive IBCs was 91.76 ± 3.53% in the apical turn.

At P15, the Lgr6-EGFP expression in the middle and basal turns was similar to the expression at P10 (*n* = 4) (Figures 4, 5, 6D,E, Tables 2, 3). The percentage of Lgr6-EGFP-positive IPCs was 14.21 ± 3.43% and 70.32 ± 5.12% in the middle and basal turns, respectively. The percentage of Lgr6-EGFP-positive IBCs was 99.79 ± 3.56% and 98.34 ± 4.12% in the middle and basal turns, respectively. In the apical turn, Lgr6-EGFP was expressed only in the IBCs (*n* = 4) (Table 3), and the percentage of Lgr6-EGFP-positive IBCs was 98.12 ± 4.1%.

### Lgr6-EGFP expression is only seen in IBCs at P20 and completely disappears at P30

At P20, Lgr6-EGFP was expressed only in the IBCs, not in the IPCs, in all three turns. The Lgr6 expression level decreased gradually from the basal to apical turn (n = 4) (Figure 6 and Table 3). The percentage of Lgr6-EGFP-positive IBCs was 19.23 ± 3.49%, 59.43 ± 4.23%, and 90.67 ± 5.01% in the apical, middle, and basal turns, respectively. At P30, we did not detect any Lgr6-EGFP expression in any cells in any of the turns (*n* = 4) (Figure 6).

### Heterozygous Lgr6-EGFP-Ires-CreERT2 mice have normal hearing

Heterozygous Lgr6-EGFP-Ires-CreERT2 mice have been reported to be healthy (Snippert et al., 2010), but data about their auditory function have not yet been provided. According to our ABR measurements, there was no significant threshold difference between the heterozygous Lgr6-EGFP-Ires-CreERT2 mice and their age-matched wild-type littermates at P30 (One-Way ANOVA, *P* = 0.79) (Figure 6C).

### Wnt signaling is required to maintain the expression of Lgr6

**Figure 7:**
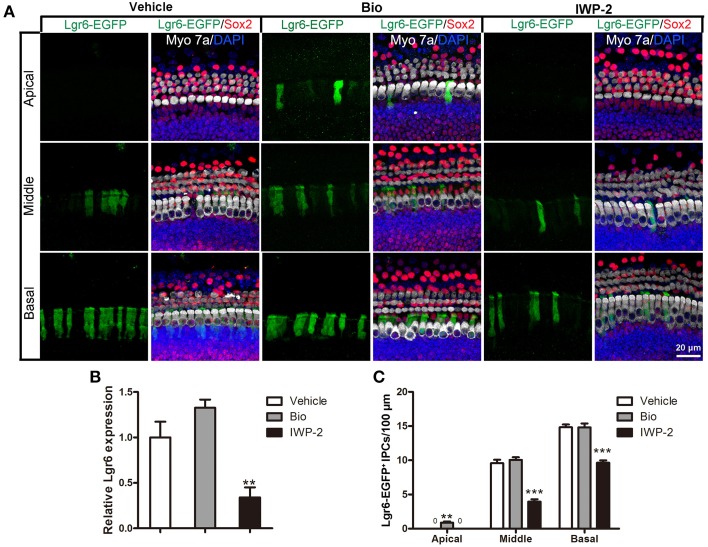
**Wnt signaling regulates the expression of Lgr6. (A)** Bio treatment significantly increased Lgr6-EGFP expression in the apical turn, but the Lgr6-EGFP expression level did not change in the middle and basal turns compared with the PBS vehicle group. IWP-2 treatment significantly decreased Lgr6-EGFP expression in both the middle and basal turns. **(B)** Quantitative PCR results showed Bio treatment slightly increased *Lgr6* expression and IWP-2 treatment significantly decreased *Lgr6* expression. **(C)** The quantification of Lgr6-EGFP-positive inner pillar cells per 100 μm cochlear epithelium. **p < 0.01, ***p < 0.001.

To investigate whether Wnt signaling can regulate Lgr6 expression, we dissected out the cochlea from P3 Lgr6-EGFP-Ires-CreERT2 mice and treated the cochlear explants with the Wnt agonist Bio (5 μM) or the Wnt antagonist IWP-2 (25 μM) for 3 days. DMSO treatment was the vehicle control. In control cochleae, there were no Lgr6-EGFP-positive cells in the apical turn. When treated with the Wnt agonist Bio, the number of Lgr6-EGFP-positive cells increased significantly in the apical turn (*n* = 4, *P* < 0.01) (Figures 7A,C and Table 4); however, the Lgr6-EGFP-positive cell numbers in the middle and basal turns did not change significantly compared to controls (*n* = 4, *P* = 0.25, and *P* = 0.95, respectively) (Figures 7A,C and Table 4). When treated with the Wnt antagonist IWP-2, the Lgr6-EGFP-positive cell number decreased significantly both in the middle and basal turns compared to controls (*n* = 4, *P* < 0.01) (Figures 7A,C and Table 4). qPCR experiments showed similar results. We found that Bio treatment increased the Lgr6 expression level, but the increase was not statistically significant (*n* = 4, *P* = 0.76) (Figure 7B), while IWP-2 significantly decreased the Lgr6 expression level (*n* = 4, *P* < 0.01) (Figure 7B). These results suggested that Wnt signaling is required to maintain the expression of Lgr6 *in vitro*.

**Table 4:** **Quantification of Lgr6-EFGP/Sox2 double-positive inner pillar cells per 100 μm cochlear length after Wnt agonist/antagonist treatment**.

**Treatment**	**Apical**	**Middle**	**Basal**
Vehicle	0	1.05 ± 0.39	14.80 ± 0.88
Bio	0.88 ± 0.18	10.67 ± 0.25	14.03 ± 0.37
IWP-2	0	3.95 ± 0.36	9.65 ± 0.33

*All the data are shown as the average ± S.D. n = 4 for all the samples*.

### Lgr6-positive cells isolated by flow cytometry can generate hair cells *in vitro*

**Figure 8:**
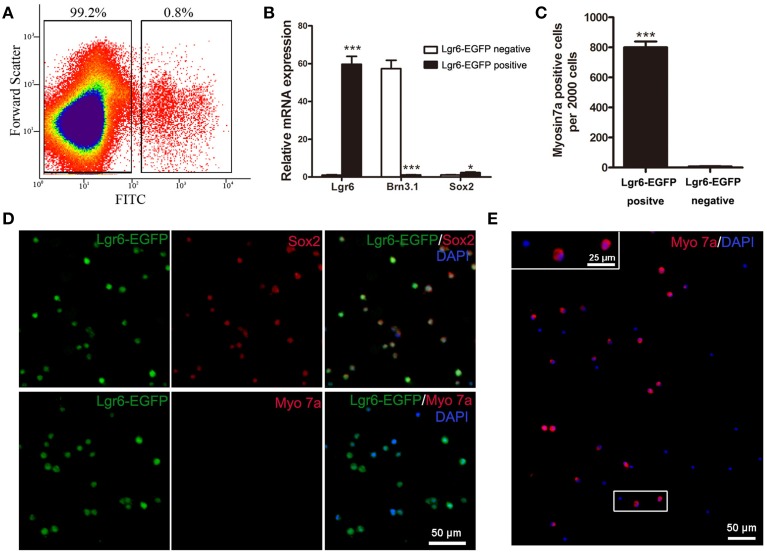
**Flow cytometry-isolated Lgr6-positive cells can generate hair cells *in vitro*. (A)** Lgr6-GFP-positive and Lgr6-GFP-negative cells were isolated using flow cytometry. **(B)** Quantitative PCR showed that isolated Lgr6-EGFP-positive cells had significantly higher Lgr6 expression, slightly higher Sox2 expression, and significantly lower Brn3.1 expression compared to the Lgr6-EGFP-negative cells. **(C)** Isolated Lgr6-EGFP-positive cells generated significantly more myosin 7a-positive cells than Lgr6-EGFP-negative cells. **(D)** Immunostaining showed that among the hair cells that developed from isolated Lgr6-EGFP-positive cells, 95% were Lgr6-EGFP-positive, 96% were Sox2-positive, and none were myosin 7a-positive. **(E)** Isolated Lgr6-GFP-positive cells can generate myosin 7a+ hair cells after 10 days of *in vitro* culture. *p < 0.05, ***p < 0.001.

To further characterize the Lgr6-positive cells, we isolated these cells with FACS (florescence-activated cell sorting) and cultured them to test whether the Lgr6-positive cells could generate hair cells *in vitro*. In this experiment, we dissected out the cochlea from 40 to 50 Lgr6-EGFP-Ires-CreERT2 mice and purified the Lgr6-EGFP-positive cells by FACS. We found that around 0.8% of the cells were Lgr6-EGFP-positive (*n* = 3) (Figure 8A). Immunohistochemical staining showed that 94.43 ± 3.67% of the Lgr6-positive cells sorted by flow cytometry were also EGFP positive (*n* = 3). Isolated Lgr6-EGFP-positive cells showed no staining for the hair cell marker myosin 7a (*n* = 3) (Figure 8D), but almost all cells were positive for the supporting cell marker Sox2 (95.65 ± 44.35%, *n* = 3) (Figure 8D). qPCR data also showed that the Lgr6-EGFP-positive cells had significantly higher Lgr6 expression (*P* < 0.001), slightly higher Sox2 expression (*P* < 0.05), and significantly lower hair cell marker Brn3.1 expression (*P* < 0.001) compared to the EGFP-negative cells (*n* = 3) (Figure 8B). We then cultured the flow cytometry-isolated Lgr6-EGFP-positive cells at a density of 20 cells/μL for 10 days and found that these Lgr6-EGFP-positive cells could generate myosin 7a-positive hair cells. After 10 days of culture, 2000 Lgr6-EGFP-positive cells could generate 802 ± 27 myosin 7a-positive hair cells. In contrast, 2000 Lgr6-negative cells could only generate around 9 ± 2 myosin 7a-positive hair cells (*n* = 3) (Figures 8C,E). This result indicated that Lgr6-positive cells might also have the ability to serve as hair cell progenitors.

## Discussion

In this study, we used Lgr6-EGFP-Ires-CreERT2 knock-in mice to determine the expression pattern of the *Lgr6* gene. During embryonic development, Lgr6 was first observed at E15.5 in one row of Sox2-positive progenitor cells. We observed the highest level of expression in the basal turn of the organ of Corti followed by reduced expression in the middle turn and no expression in the apical turn. Lgr6 expression in the IPCs increased from E15.5 to P1 at which time it began to decrease. From P7 to P15, we observed Lgr6-EGFP expression not only in the IPCs but also in the IBCs. From P7 to P15, the Lgr6 expression level gradually decreased in the IPCs and gradually increased in the IBCs. At P20, Lgr6 expression in the IPCs disappeared and Lgr6 expression was only observed in the IBCs with a gradient from the basal to apical turns. At P30, Lgr6 expression had completely disappeared in all three turns.

The *Lgr6* gene is homologous to *Lgr5*. Previous studies reported that *Lgr5* is expressed in the prosensory region during embryonic development and is expressed in the third row of Deiters' cells, the IPCs, the medial inner phalangeal cells, and the lateral GER at P3. From P3 to P7, the level of Lgr5-EGFP expression gradually decreased in the IPCs, the inner phalangeal cells, and the lateral GER. At P15, only the third row of Deiters' cells expressed Lgr5-EGFP (Chai et al., 2011; Shi et al., 2012). In this study, when we compared the gene expression of *Lgr6* and *Lgr5*, we found different expression patterns during the development and maturation of the organ of Corti. Generally speaking, during embryonic development Lgr6 is expressed in a sub-population of Lgr5-positive prosensory cells, and we observed Lgr6 expression in the Lgr5-positive IPCs from E18.5 to P3. From P7 to P15, Lgr5 expression in the IPCs, the medial inner phalangeal cells, and the lateral GER almost completely disappeared and Lgr5 expression was restricted to the third row of Deiters' cell. However, at the same developmental stage Lgr6 was expressed in both the IPCs and IBCs. At P20, Lgr5 was restricted to the third row of Deiters' cells and Lgr6 was restricted to the IBCs. At P30, Lgr5 was still expressed in the third row of Deiters' cells, but Lgr6 expression completely disappeared. The other difference between Lgr5 and Lgr6 expression is the expression pattern along the apical—basal axis. Lgr6 expression had a clear gradient from the basal to apical turns, and prior to P7 Lgr6 expression was restricted to the middle and basal turns and no Lgr6 was expressed in the apical turn. However, Lgr5 was expressed in all three turns with no clear difference between the turns.

The canonical Wnt/β-catenin signaling pathway is known to play crucial roles in cochlear duct development by regulating proliferation and hair cell differentiation (Jacques et al., 2012; Shi et al., 2014). Wnt/β-catenin signaling regulates the expression of downstream target genes, including *Lgr5* and *Axin2*, and modulates the proliferation of Lgr5-positive and Axin2-positive progenitor cells (Chai et al., 2011, 2012; Shi et al., 2012, 2013). In cell cultures, the Wnt agonist Wnt3a increases the *Lgr5* mRNA expression *in vitro*, and exogenous Wnt3a prevents the decline of Lgr5-EGFP expression in cultured P6 Lgr5-EGFP cochleae. In addition, the Wnt antagonist Fz8CRD decreases *Lgr5* mRNA expression *in vitro*; in P3 Lgr5-EGFP cochleae, Lgr5-EGFP expression cannot be detected when treated with Fz8CRD, suggesting Lgr5-EGFP expression is dependent on active Wnt signaling (Chai et al., 2011). In this study, we investigated the effect of Wnt/β-catenin signaling on another Wnt downstream gene, *Lgr6*. We found that activating Wnt/β-catenin signaling with the exogenous Wnt agonist Bio significantly increased Lgr6 expression in the apical turn, but Lgr6 expression in the middle and basal turns was not significantly changed. On the other hand, inhibition of Wnt/β-catenin signaling with Iwp2 significantly reduced Lgr6 expression in the cochleae suggesting that Wnt signaling is required to maintain the expression of Lgr6.

Lgr6 has been reported to be a stem cell marker in numerous organs, including the skin, taste buds, and lungs (Snippert et al., 2010; Oeztuerk-Winder et al., 2012; Ren et al., 2014). In hair follicles, Lgr6 is expressed in a stem cell population above the follicle bulge, and these Lgr6-positive stem cells have the ability to generate all cell lineages found in the skin (Snippert et al., 2010). In taste buds, both Lgr5 and Lgr6 mark the stem cells, and the individually Lgr5-positive or Lgr6-positive cells can regenerate functional taste cells *ex vivo* (Ren et al., 2014). In the inner ear, previous studies have demonstrated that the Wnt downstream target gene *Lgr5* marks the inner ear progenitor cells and the Lgr5-positive cell can generate hair cells both *in vitro* and *in vivo*. After hair cell damage, these Lgr5-positive progenitors also have the ability to regenerate new hair cells via both mitotic regeneration and direct differentiation (Chai et al., 2012; Shi et al., 2012, 2013; Jan et al., 2013; Cox et al., 2014). In this study, we used Lgr6-EGFP-Ires-CreERT2 mice and flow cytometry to characterize the identity of Lgr6-positive cells. We found that Lgr6-positive cells isolated by FACS also have the ability to differentiate into hair cells *in vitro*, and this suggests that Lgr6 might also mark hair cell progenitors in the organ of Corti. The detailed identity of the Lgr6-positive cells and the mechanisms involved in regulating these cells are worthy of study in the future.

In summary, we have determined the detailed expression pattern of the Wnt downstream target gene *Lgr6* in the mouse cochlear duct from embryonic to adult ages, and we demonstrated that Wnt signaling is required to maintain the expression of Lgr6 in whole organ cultures *in vitro*. In addition, we demonstrated that when isolated by FACS, Lgr6-positive cells can differentiate into hair cells *in vitro*, indicating that Lgr6-positive cells might have the potential to be inner ear hair cell progenitors. These findings suggest that the Lgr6-positive cells might also serve as candidate therapeutic targets for hair cell regeneration.

## Author contributions

Conceived and designed the experiments: HL. Performed the experiments: YZ, YC, LX, and LL. Analyzed the data: YZ, YC, and HL. Wrote the paper: YZ, YC, and HL.

### Conflict of interest statement

The authors declare that the research was conducted in the absence of any commercial or financial relationships that could be construed as a potential conflict of interest.

## Supplementary material

Figure S1 **At P3, Lgr6-EGFP was expressed only in the inner border cells in the middle and basal turns, and there was no Lgr6-EGFP expression in the apical turn**.
