# Supplementary figures and images for: Dynamic expression of Lgr6 in the developing and mature mouse cochlea
Source: Front Cell Neurosci. 2015 May 12;9:165. doi: 10.3389/fncel.2015.00165 (PMC4428082; doi:10.3389/fncel.2015.00165)

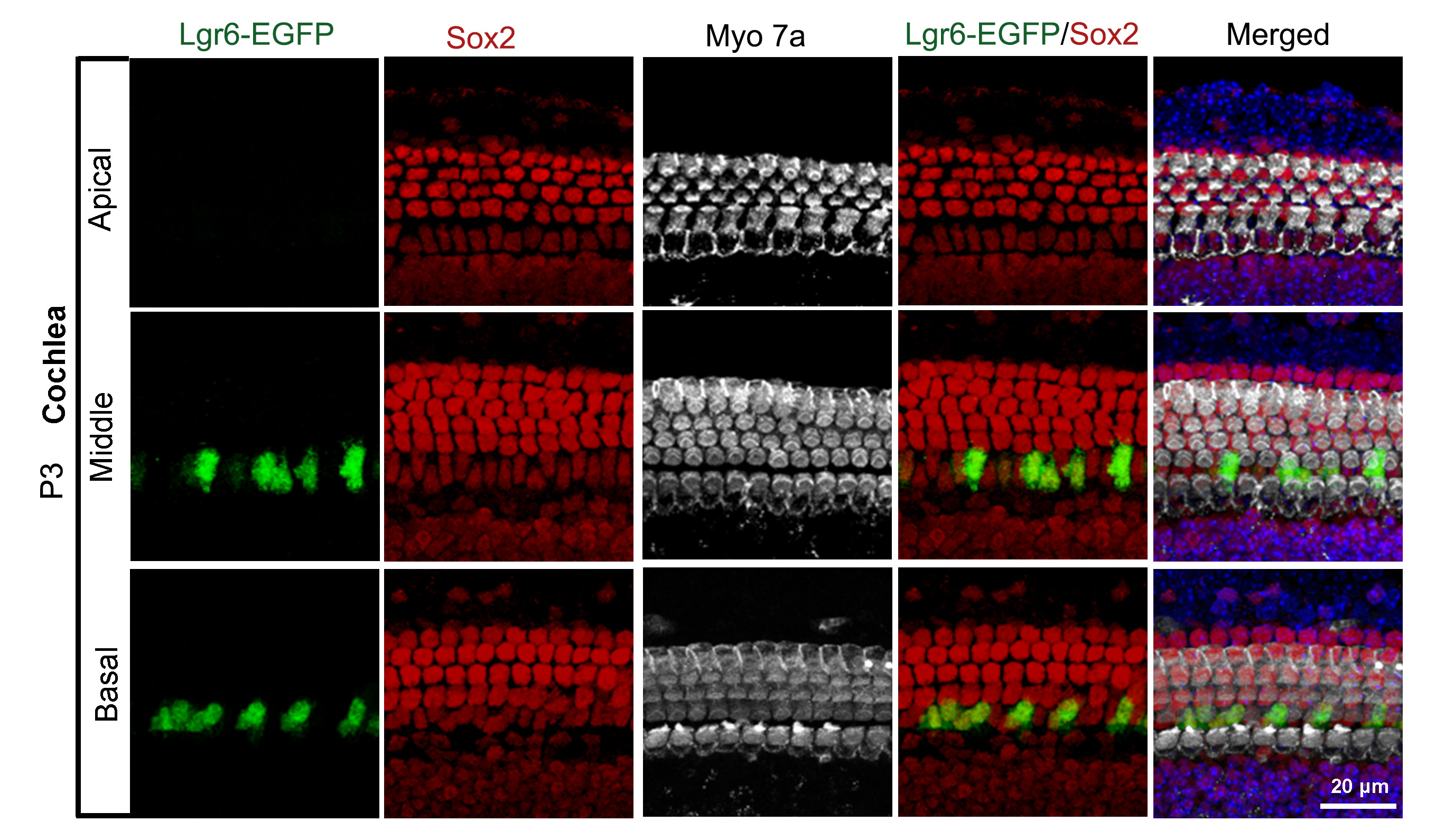

Supplement: Figure S1 — At P3, Lgr6-EGFP was expressed only in the inner border cells in the middle and basal turns, and there was no Lgr6-EGFP expression in the apical turn. [file Image1.JPEG]
